# The emergence, implementation, and future growth of pharmacogenomics in psychiatry: a narrative review

**DOI:** 10.1017/S0033291723002817

**Published:** 2023-12

**Authors:** Chad A. Bousman, Abdullah Al Maruf, Diogo Ferri Marques, Lisa C. Brown, Daniel J. Müller

**Affiliations:** 1 The Mathison Centre for Mental Health Research & Education, Hotchkiss Brain Institute, University of Calgary, Calgary, AB, Canada; 2 Department of Psychiatry, University of Calgary, AB, Canada; 3 Department of Medical Genetics, University of Calgary, Calgary, AB, Canada; 4 Departments of Physiology and Pharmacology, and Community Health Sciences, University of Calgary, Calgary, AB, Canada; 5 AB Children's Hospital Research Institute, University of Calgary, Calgary, AB, Canada; 6 Department of Psychiatry, University of Melbourne, Melbourne, VIC, Australia; 7 College of Pharmacy, Rady Faculty of Health Sciences, Winnipeg, MB, Canada; 8 Great Scott! Consulting, New York, NY, USA; 9 Pharmacogenetics Research Clinic, Campbell Family Mental Health Research Institute, Centre for Addiction and Mental Health, Toronto, ON, Canada; 10 Department of Psychiatry, University of Toronto, Toronto, ON, Canada; 11 Department of Psychiatry, Psychosomatics and Psychotherapy, Center of Mental Health, University Hospital of Wurzburg, Wurzburg, Germany

**Keywords:** antidepressants, antipsychotics, implementation, mental health, pharmacogenomics, precision medicine, psychiatry, psychopharmacology, psychotropics

## Abstract

Psychotropic medication efficacy and tolerability are critical treatment issues faced by individuals with psychiatric disorders and their healthcare providers. For some people, it can take months to years of a trial-and-error process to identify a medication with the ideal efficacy and tolerability profile. Current strategies (e.g. clinical practice guidelines, treatment algorithms) for addressing this issue can be useful at the population level, but often fall short at the individual level. This is, in part, attributed to interindividual variation in genes that are involved in pharmacokinetic (i.e. absorption, distribution, metabolism, elimination) and pharmacodynamic (e.g. receptors, signaling pathways) processes that in large part, determine whether a medication will be efficacious or tolerable. A precision prescribing strategy know as pharmacogenomics (PGx) assesses these genomic variations, and uses it to inform selection and dosing of certain psychotropic medications. In this review, we describe the path that led to the emergence of PGx in psychiatry, the current evidence base and implementation status of PGx in the psychiatric clinic, and finally, the future growth potential of precision psychiatry via the convergence of the PGx-guided strategy with emerging technologies and approaches (i.e. pharmacoepigenomics, pharmacomicrobiomics, pharmacotranscriptomics, pharmacoproteomics, pharmacometabolomics) to personalize treatment of psychiatric disorders.

## Introduction

For individuals with moderate-to-severe psychiatric disorders, pharmacotherapies are considered part of first-line treatment options. Unfortunately, finding an efficacious and tolerable pharmacotherapy for everyone is often a clinically challenging process that can take months to years using available treatment protocols. This challenge, in part, is underpinned by interindividual variation in efficacy and tolerability of commonly used psychotropic drugs (e.g. antidepressants, antipsychotics) and the realization that treatment protocols that work at the population level are not necessarily suitable at the individual level. For example, randomized controlled trial findings have shown about one-third of individuals with major depressive disorder (MDD) do not achieve symptom remission when treated according to the Sequenced Treatment Alternatives to Relieve Depression (STAR*D) protocol (Rush et al., 2006). As such, there is a need for additional prescribing strategies that can augment current treatment protocols and boost effective medication management of psychiatric disorders. Pharmacogenomics (PGx) (i.e. the study and use of an individual's genomic information to predict response to medications) is one such strategy that has gained significant momentum and is fueling the precision psychiatry movement (Maruf & Bousman, 2022).

The PGx-guided prescribing strategy works by leveraging variation in genes that are involved in pharmacokinetic (i.e. absorption, distribution, metabolism, elimination) and pharmacodynamic (e.g. receptors, signaling pathways) processes that in large part, determine whether a medication will be efficacious or tolerable. For certain psychotropic medications (e.g. psychostimulants), these processes have not been fully elucidated, whereas for other medications (e.g. antidepressants, antipsychotics), robust (replicated) associations between genomic variants and exposure (blood concentrations) as well as clinical outcomes (e.g. adverse drug events, symptom reduction) are established and ready for implementation (Bousman et al., 2021). To date, variants in genes that encode proteins within the human leukocyte antigen (HLA) family and drug metabolizing enzymes within the cytochrome P450 (CYP) family have the most robust evidence, particularly two members of the HLA family (*HLA-A* and *HLA-B*) and three members of the CYP family (*CYP2C19*, *CYP2D6*, and *CYP2B6*). In this review, we describe the path that led to the emergence of PGx in psychiatry, the current evidence base and implementation status of PGx in the psychiatric clinic, and finally, the future growth potential of precision psychiatry via the convergence of the PGx-guided strategy with emerging technologies and approaches to personalize treatment of psychiatric disorders.

## Emergence of PGx in psychiatry

**Figure 1.**
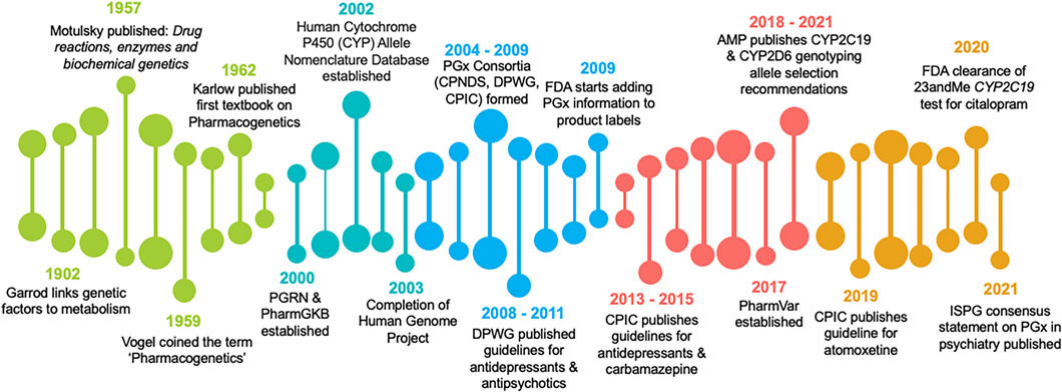
Major events in the emergence of pharmacogenomics in psychiatry. AMP, Association of Molecular Pathology; CPIC, Clinical Pharmacogenetics Implementation Consortium; CPNDS, Canadian Pharmacogenomics Network for Drug Safety; DPWG, Dutch Pharmacogenetics Working Group; FDA, Food and Drug Administration; ISPG, International Society of Psychiatric Genetics; PGRN, Pharmacogenomics Global Research Network; PGx, pharmacogenomic; PharmGKB, Pharmacogenomics Knowledge Base; PharmVar, Pharmacogene Variation Consortium.

The events leading to the emergence of PGx in psychiatry began in ancient Greece and has been described in detail elsewhere (Ampong, 2019; Müller & Rizhanovsky, 2020). In brief, the Greek scientist Pythagoras described an adverse reaction, now known as acute hemolytic anemia, that was experienced by some, but not all, people who consumed fava beans. Although at that time Pythagoras was unaware that genetic variants in the gene encoding glucose-6-phosphate dehydrogenase (G6PD) caused this adverse reaction, it is often considered the first PGx observation (Meletis, 2012). However, the foundation for modern PGx was not established until the twentieth century, when the seminal works of Sir Archibald Garrod (Prasad & Galbraith, 2005), Arno Motulsky (Motulsky, 1957), Friedrich Vogel (Vogel, 1959), and Werner Kalow (Kalow, 1962) were published. These works coupled with the completion of the Human Genome project in 2003 facilitated a series of key events that led to the current application of PGx in psychiatry (Fig. 1).

Among the most influential of these key events was the establishment of PGx implementation consortia, such as the Clinical Pharmacogenetics Implementation Consortium (CPIC) and the Dutch Pharmacogenetics Working Group (DPWG). These consortia were formed to facilitate the use of PGx testing for patient care via development of evidence-based, peer-reviewed, and freely available clinical prescribing guidelines. The first PGx-based dosing guidelines relevant to psychiatry were published in 2008 by the DPWG and included dosing recommendations for antidepressants (i.e. clomipramine, imipramine, nortriptyline, paroxetine, and venlafaxine), antipsychotics (i.e. haloperidol, risperidone, and zuclopenthixol), and one medication (i.e. atomoxetine) for attention-deficit hyperactivity disorder (ADHD) based on *CYP2D6* genotype (Swen et al., 2008). In 2011, these guidelines were updated, and dosing recommendations were included for additional antidepressants (i.e. amitriptyline, citalopram, doxepin, escitalopram, and sertraline) and antipsychotics (i.e. aripiprazole, fluphenthixol) based on *CYP2C19* or *CYP2D6* genotype (Swen et al., 2011). CPIC followed with dosing guidelines of their own for tricyclic antidepressants (Hicks et al., 2013) and carbamazepine (Leckband et al., 2013) in 2013 as well as a guideline for selective serotonin reuptake inhibitors in 2015 (Hicks et al., 2015) and atomoxetine in 2019 (Brown et al., 2019). Updates to the CPIC tricyclic antidepressant, carbamazepine/oxcarbazepine, and serotonin reuptake inhibitor antidepressant guidelines were published in 2016 (Hicks et al., 2016), 2018 (Phillips et al., 2018), and 2023 (Bousman et al., 2023), respectively. In 2023, the DPWG again updated their antipsychotic prescribing guidelines, adding additional dosing recommendations for brexpiprazole and pimozide based on *CYP2D6* genotype and quetiapine based on *CYP3A4* genotype (Beunk et al., 2023). A CPIC antipsychotic dosing guideline is anticipated in 2024.

To date, CPIC and DPWG have collectively developed PGx-based dosing guidelines for 14 antidepressants, seven antipsychotics, three anticonvulsant/mood stabilizers, and one medication for ADHD (Table 1). Importantly, these guidelines have been endorsed by the American Society of Health-System Pharmacists (ASHP, n.d.), American Society for Clinical Pharmacology and Therapeutics (ASCPT, n.d.), Canadian Paediatric Society (Canadian Paediatric Society, 2023), as well as six European healthcare organizations including the European Association for Clinical Pharmacology and Therapeutics and the European Association of Hospital Pharmacists (‘Statement of support for the use of European pharmacogenomic guidelines,’, n.d.). Moreover, in 2020, the US Food and Drug Administration (FDA) issued clearance for 23andMe to offer *CYP2C19* testing for citalopram without the need for confirmatory testing by an additional certified clinical laboratory, marking the first FDA clearance of a PGx test for a psychotropic medication (Food & Drug Administration, 2020). Finally, an expert group convened by the International Society of Psychiatric Genetics came to a consensus that the current published evidence supports the use of PGx testing for *CYP2D6* and *CYP2C19* to inform dosing of antidepressants, antipsychotics, and atomoxetine, as well as testing for *HLA-A* and *HLA-B* prior to initiation of carbamazepine or oxcarbazepine (Bousman et al., 2021).

**Table 1.** Drug–gene pairs with pharmacogenomic prescribing guidelines

Class	Drug(s)	Gene(s)	Actionable genotype-predicted phenotype(s)	Recommendation summary^a^	Source	Level of evidence^b^
Antidepressants	Citalopram	CYP2C19	UM or PM	Select alternative drug	CPIC	1A
	Escitalopram	CYP2C19	UM or PM	Select alternative drug	CPIC	1A
	Fluvoxamine	CYP2D6	PM	25–50% lower starting dose, slower titration	CPIC	1A
	Paroxetine	CYP2D6	UM	Select alternative drug	CPIC	1A
			IM	Lower starting dose, slower titration		
			PM	50% lower starting and maintenance dose, slower titration		
	Sertraline	CYP2C19-CYP2B6	UM-UM; UM-RM; RM-UM	Higher maintenance dose or select alternative drug	CPIC	1A
			NM-IM; IM-NM; IM-PM	Slower titration and lower maintenance dose		
			NM-PM	Lower starting dose, slower titration, 25% lower maintenance dose		
			PM-UM; PM-NM; PM-IM	Lower starting dose, slower titration, 50% lower maintenance dose		
			PM-PM	Select alternative drug		
	Desipramine	CYP2D6	UM or PM	Select alternative drug	CPIC	1A
			IM	25% lower starting dose		
	Nortriptyline	CYP2D6	UM or PM	Select alternative drug	CPIC	1A
			IM	25% lower starting dose		
	Amitriptyline, clomipramine, doxepin, imipramine, trimipramine	CYP2C19-CYP2D6	UM-UM; UM-NM; UM-IM; UM-PM; RM-UM; RM-NM; RM-IM, RM-PM; NM-UM; NM-PM; IM-UM; IM-PM; PM-UM; PM-NM, PM-IM; PM-PM	Select alternative drug	CPIC	1A
			NM-IM; IM-NM	25% lower starting dose		
	Venlafaxine	CYP2D6	PM	Select alternative drug	CPIC	1A
	Vortioxetine	CYP2D6	UM	Select alternative drug	CPIC	1A
			PM	50% lower starting, titrate to max dose of 10 mg		
Antipsychotics	Aripiprazole	CYP2D6	PM	Administer no more than 10 mg/day	DPWG	1A
	Brexpiprazole	CYP2D6	PM	50% of normal dose	DPWG	1A
	Risperidone	CYP2D6	UM	Select alternative drug	DPWG	1A
			PM	67% of normal dose		
	Quetiapine	CYP2D6	PM	Select alternative drug or 30% of normal dose	DPWG	1A
	Haloperidol	CYP2D6	UM	1.5 times the normal dose or select alternative	DPWG	1A
			PM	60% of normal dose		
	Pimozide	CYP2D6	IM	80% of the normal dose	DPWG	1A
			PM	50% of the normal dose		
	Zuclopenthixol	CYP2D6	IM	75% of normal dose	DPWG	1A
			PM	50% of normal dose		
ADHD	Atomoxetine	CYP2D6	UM	Select alternative drug	DPWG	1A
			IM or PM	Lower maintenance dose		
Anticonvulsant/mood stabilizers	Carbamazepine	HLA-A - HLA-B	Carrier of A*31:01 or B*15:02	Select alternative drug	CPIC	1A
	Oxcarbazepine	HLA-B	Carrier of B*15:02	Select alternative drug	CPIC	1A

IM, intermediate metabolizer; NM, normal metabolizer; PM, poor metabolizer; RM, rapid metabolizer; UM, ultrarapid metabolizer.

a Recommendations are current as of 1 June 2023. Full recommendation, evidence reviews, and updates can be accessed via the Pharmacogenomics Knowledge Base: https://www.pharmgkb.org/

b Information on assignment of level of evidence can be found here: https://www.pharmgkb.org/page/clinAnnLevels

## Implementation of PGx testing in psychiatry

Despite the availability of PGx-based dosing guidelines, inclusion of PGx information in certain FDA-approved product labels, and endorsements from professional societies, the adoption of PGx testing into routine mental health care has been modest. On the Innovation Adoption Curve (Rogers, 1981), PGx in psychiatry is in the ‘early adopter’ phase and approaching the ‘chasm’, where innovations either fail or succeed in gaining the traction required to cross into the ‘early majority’ phase and achieve sustainable adoption. Several hurdles to crossing the chasm have been identified and reviewed in detail elsewhere, including (1) uncertainty about the clinical efficacy and cost-effectiveness (Maruf & Bousman, 2022; Morris et al., 2022; Murphy, Fonseka, Bousman, & Müller, 2022), (2) low perceived clinical generalizability (Bousman et al., 2021), (3) minimal standardization and regulation (Bousman & Dunlop, 2018; Bousman, Jaksa, & Pantelis, 2017; Fan & Bousman, 2019), (4) lack of incorporation into clinical practice guidelines (Maruf & Bousman, 2022), (5) challenges with integration into electronic health/medical records (Caraballo et al., 2020; Gammal, Berenbrok, Empey, & Massart, 2021), (6) high testing costs and inequitable reimbursement (Empey, Pratt, Hoffman, Caudle, & Klein, 2021), and (7) gaps in end user knowledge and education (Bousman et al., 2022b; Hayashi & Bousman, 2022; Jameson et al., 2021; Jessel, Al Maruf, Oomen, Arnold, & Bousman, 2022; Soda et al., 2023). These hurdles, however, are not insurmountable.

To overcome these hurdles, we recommend: (1) real-world and controlled studies of clinical effectiveness and cost effectiveness to understand clinical and economic benefits of PGx, (2) investment in discovery of additional PGx markers, particularly those related to pharmacodynamic processes, to expand the number of drugs with actionable recommendations, more precisely identify suitable medication options, and improve generalizability in diverse populations, (3) involvement of government or clinical societies in the development and implementation of PGx testing standards via a national or international certification process, (4) partnerships between PGx expert groups (e.g. CPIC) and professional societies that develop clinical practice guidelines to identify opportunities to integrate PGx evidence, (5) prioritization by electronic health/medical record manufactures to improve their product design to seamlessly integrate and present PGx data across systems, (6) encourage health care payors to adopt reimbursement strategies that ensure equitable access to PGx testing, and (7) investment in the development and maintenance of PGx educational materials for healthcare trainees, advanced clinicians, pharmacists, genetic counselors, and the community.

Exemplars of successful and sustainable PGx testing implementation exist (Duarte et al., 2021; Luczak et al., 2021). In 2004, Cincinnati Children's Hospital Medical Center became the first to offer PGx testing in psychiatry and today, all patients admitted to the inpatient psychiatry service receive PGx testing as part of routine care (Ramsey et al., 2018). They were joined by other PGx innovators such as St Jude Children's Research Hospital (Hoffman et al., 2014), Mayo Clinic (Bielinski et al., 2014), Vanderbilt University Medical Center (Van Driest et al., 2014), University of Florida Health Personalized Medicine Program (Cavallari et al., 2017), and several other institutions in the Implementing Genomics in Practice Network (Sperber et al., 2017) and Ubiquitous Pharmacogenomics Consortium (Blagec et al., 2018). These innovators, in part, have created a bridge by which PGx-guided prescribing in psychiatry can successfully cross the chasm. As of March 2023, at least 70 institutions across the globe are implementing PGx-guided prescribing and most include psychiatric indications (CPIC, 2019).

In parallel with the innovative efforts of academic and healthcare institutions, commercial PGx laboratories have also played a role in the clinical implementation of PGx in psychiatry. In 2006, Assurex Health (now Myriad Neuroscience) was founded with patented technology licensed from Cincinnati Children's Hospital Medical Center and Mayo Clinic, marking the launch of the first commercial PGx testing laboratory focused on psychotropic drugs (Assurex Health, 2016). Since then, numerous commercial PGx laboratories have emerged (Bousman & Hopwood, 2016; Maruf et al., 2020) and some medical centers have partnered with these laboratories to provide PGx testing for their patients (RPRD Diagnostics, 2017; St. Catherine Specialty Hospital, n.d.). To date, PGx tests offered by commercial laboratories have been the focus of most PGx clinical trials in psychiatry. For example, 11 of the 13 clinical trials that have examined the efficacy of PGx testing to inform prescribing of antidepressants for patients with depression were supported by commercial PGx testing laboratories (Brown et al., 2022). Pooled results from these clinical trials showed patients receiving PGx-guided antidepressant treatment were 41% (95% CI 15–74%) more likely to achieve symptom remission relative to their counterparts that received treatment as usual (Brown et al., 2022). However, these encouraging trial findings have been tempered by concerns within the academic and clinical community about industry bias, the use of inappropriate comparison groups, and unsatisfactory intervention blinding (Smith & Nemeroff, 2020). Additional concerns have been raised by the FDA related to claims made by commercial PGx testing laboratories, resulting in the issuance of a warning letter to Inova Genomics Laboratory in 2019 (FDA, 2019) and two safety communications cautioning the use of PGx testing results that are not based on FDA-approved product labeling (Ellingrod, 2019).

To address the critiques from the academic and clinical community, several investigator-initiated clinical trials have been launched (trial registration numbers: NCT03749629, NCT04623099, NCT04445792, ACTRN12621000181808, ACTRN12621001374853). Moreover, the FDA has become more proactive in the PGx space. In February of 2020, the FDA released and has regularly updated a ‘Table of Pharmacogenetic Associations’ that lists gene–drug pairs that are ‘supported by sufficient scientific evidence’ (Food & Drug Administration, 2021) and more recently released draft guidance for pharmacogenomic data submissions to industry that will replace previous guidance published in 2005 and facilitate the use of PGx data in drug development (FDA, 2023). Unfortunately, only 39 gene–drug pairs of the 106 included in the FDA Table of Associations are included in CPIC guidelines, which has created confusion among PGx implementors and test developers (Pritchard, Patel, Stephens, & McLeod, 2022). However, it is anticipated that concordance between CPIC and the FDA will improve as communication between them is strengthened, and the evidence base continues to grow.

## Future growth of PGx in psychiatry

**Figure 2.**
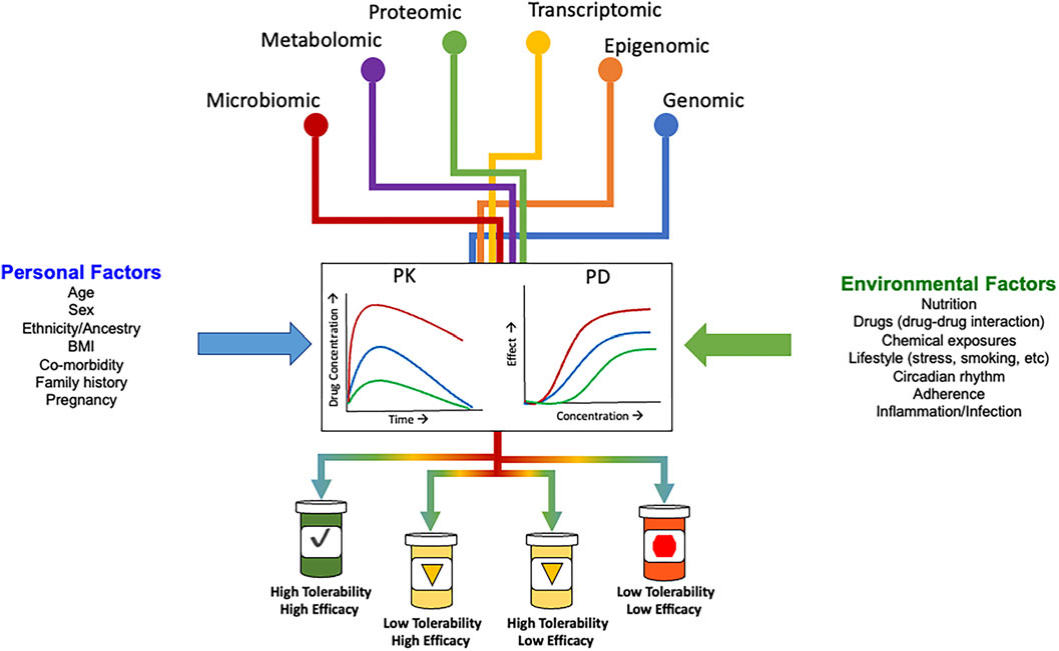
Conceptual model of precision psychotropic prescribing decision support in the future. PK, pharmacokinetic; PD, pharmacodynamic. Adapted from Maruf and Bousman (2021).

In addition to addressing the implementation hurdles discussed above, the growth of PGx-guided prescribing will, in part, hinge on investment in and integration of knowledge emerging from other pharmaco-omic approaches (e.g. polygenic scoring, pharmacoepigenomics, pharmacometabolomics, pharmacotranscriptomics, pharmacoproteomics, pharmacomicrobiomics). Below, we provide a summary of the knowledge and progress made in these other pharmaco-omic approaches. In the future, we anticipate these approaches in combination with personal and environmental factors will provide an opportunity to facilitate actionable dosing recommendations for additional psychotropic medications and refine current recommendations (Fig. 2).

The era of big data (e.g. biobanks and genome-wide association studies) has facilitated the development of tools for extracting PGx information from large genomic datasets (McInnes et al., 2021) as well as analytical strategies for interrogating the entire genome rather than individual candidate genes. On such strategy is polygenic scoring in which genetic variation across the genome is mathematically combined to derive a ‘score’ that can be used to predict or stratify a person's probability of experiencing a clinical outcome (e.g. medication response). According to a recent systematic review, 59% (30/51) of all studies using polygenic scores in PGx have been conducted within the area of mental and behavioral diseases (Johnson et al., 2022). Some of the most notable of these studies have applied this approach to antidepressant (Li, Tian, Hinds, & Team, 2020; Pain et al., 2022), clozapine (Okhuijsen-Pfeifer et al., 2022; Pardiñas et al., 2023), and lithium (Amare et al., 2023) response but results are mixed and the variance explained by polygenic scores have been modest (e.g. <10%). Although GWAS and polygenic scoring studies have shown some promise in PGx, clinical implementation is not yet warranted. Going forward, novel approaches for improving the clinical interpretability of polygenic scores are needed. One such approach is pharmagenic enrichment scores, which were developed to provide an indication of an individual's exposure to genomic variants in biological pathways with known drug targets, an approach that may be particularly useful in selection of medications for individuals with treatment-resistant conditions (Reay, Atkins, Carr, Green, & Cairns, 2020).

Pharmacoepigenomics is a field of study that explores the correlation between epigenetic changes and drug response. Epigenetics refers to heritable and potentially reversible chemical modifications (e.g. methylation, acetylation, phosphorylation) to the genome that can alter gene expression without changing the DNA sequence itself (Dupont, Armant, & Brenner, 2009). It can promote biological impacts that result in a particular phenotype that may be associated with desirable or undesirable response to medications (Micale et al., 2023). A recent review of antidepressant pharmacoepigenomics found most of the clinical research has focused on methylation of genes encoding the brain derived neurotrophic factor (*BDNF*), monoamine transporters (*SLC6A4*, *SLC6A2*), and serotonin receptors (*HTR1A*, *HTR1B*), with promising but mixed results (Hack et al., 2019). Most published antidepressant research to date are candidate-gene driven with the exception of one epigenome-wide association study (EWAS) where differential methylation at CpG sites upstream of the *CHN2* (encodes beta-chimerin, a protein that is important for cell proliferation) and *JAK2* (encodes anus kinase 2, a protein that promotes the growth of cells) transcriptional start site regions were identified as potential predictors of antidepressant response (Ju et al., 2019). Clinical studies are also encouraging but mixed for antipsychotic pharmacoepigenomics, which have primarily assessed methylation changes in candidate genes linked to the serotonergic (e.g. *SLC6A4*, *HTR1A*, *HTR2A*) and dopaminergic (e.g. *DRD2*) systems (Ovenden, McGregor, Emsley, & Warnich, 2018). In a global methylation study, hypomethylation of LINE-1 elements was associated with poor response to risperidone treatment in patients with schizophrenia (Marques et al., 2020). In another whole-genome DNA methylation study, six genes (*APIS3*, *C16orf59*, *KCNK15*, *LOC146336*, *MGC16384*, and *XRN2*) were found to be hypermethylated and were identified as good markers of treatment-induced effects in male schizophrenia patients before and after achieving complete remission (Rukova et al., 2014). Despite these promising findings, pharmacoepigenomic-guided psychotropic prescribing is not ready for clinical implementation, although one can imagine it will contribute to future prescribing decision-making tools. The shift to whole epigenome approaches that not only capture methylation but also other epigenomic mechanisms (e.g. histone modifications) will undoubtedly expedite the identification of informative pharmacoepigenomic profiles and their use in guiding psychotropic treatment.

Pharmacomicrobiomics, the study of how the gut microbiome interacts with medication, is another emerging approach for understanding psychotropic efficacy and tolerability (Cussotto, Clarke, Dinan, & Cryan, 2019; Generoso, Giridharan, Lee, Macedo, & Barichello, 2021; Brown, Bobo, Gall, Muller, & Bousman, 2023). Gut microbiota can directly alter absorption, distribution, metabolism, and elimination of psychotropics as well as indirectly modulate host cytochrome (CYP) enzymes (Collins & Patterson, 2020; Dempsey & Cui, 2019). Gut bacteria may also sequester specific medications leading to a change in the microbiome function and in some cases psychotropics can have detrimental or antimicrobial effects on the microbiome (Klünemann et al., 2021). Moreover, modification of medications by specific bacteria may result in an increase in active metabolites, which could increase response or perpetuate adverse drug reactions (Wan & Zuo, 2022; Zeng et al., 2021). Therefore, the presence of specific drug-interacting microbial species may serve as a biomarker of efficacy or tolerability to specific medications or guide modification of the microbiota to enrich beneficial species to optimize response to medications (Nita et al., 2022). Notably, the current evidence in psychiatry is primarily based on preclinical studies (Cussotto et al., 2019). However, recent work in patients with psychiatric disorders suggests that certain microbiota expression patterns are associated with treatment resistance (Thompson et al., 2023), while other patterns are associated with SSRI (Gao et al., 2023) and antipsychotic (Yuan et al., 2021) response. Furthermore, clinical trials are ongoing to understand the effect of psychotropic medications on the microbiome (NCT03414151) and the role of the microbiome in psychotropic medication response (NCT05022524). These trials, along with additional clinical studies, are expected to expand our understanding of microbiome–psychotropic drug interactions and highlight opportunities for translation into clinical care.

Pharmacotranscriptomics investigates the associations between variations in the transcriptome (i.e. the complete set of RNA molecules) and the interindividual variation in drug response. Candidate–gene expression studies have found significant associations with *SLC6A4* (serotonin transporter), *IL1β* (interleukin 1*β*), *TNF* (tumor necrosis factor alpha), and *FKBP5* (FK 506 binding protein 5) genes (reviewed in Belzeaux et al. [2018]). Whole-genome transcriptomic studies have also reported some promising results. For example, interferon regulatory factor 7 (*IRF7*) was found to be upregulated among citalopram responders in MDD patients (Mamdani et al., 2011). In another study, machine learning approaches identified and independently replicated a set of 13 transcripts that were able to predict MDD patients in symptom remission with an accuracy of 79.4% (Guilloux et al., 2015). A global gene co-expression network analysis found genes related to immune response, acute inflammatory response, and C-X-C motif chemokine ligand 8 (IL8) receptor activity to be associated with antidepressant response using three independent cohorts of patients with depression (Belzeaux et al., 2016). Although no transcriptomic markers currently have robust clinical trial evidence, the introduction of new and cheaper RNA sequencing technology will aid in transcriptomic research in psychiatry (reviewed in Maruf & Bousman [2021]).

Pharmacoproteomics is an emerging field that investigates changes in the levels of specific proteins in response to medication therapy. In psychiatry, proteomic profiling has focused on specific proteins relevant to cell communication and signaling, inflammation, and cellular growth, and maintenance in response to psychotropic drugs (reviewed in Cassoli, Guest, Santana, & Martins-de-Souza [2016]). For example, seven proteins (interleukin-16, fatty acid binding protein, ferritin, C-reactive protein, myoglobin, prolactin, and complement factor H) were found to predict the improvement in positive symptoms, and two proteins (matrix metalloproteinase 2 and insulin) were found to predict improved negative symptoms in patients with schizophrenia treated with antipsychotics (Schwarz, Guest, Steiner, Bogerts, & Bahn, 2012). In the GENDEP (genome-based therapeutic drugs for depression) study cohort, higher C-reactive protein levels were associated with better response with nortriptyline whereas lower levels were associated with better response in patients taking escitalopram (Uher et al., 2009). To date, no validated proteomic markers are available to aid clinicians in choosing a psychotropic drug for their patient.

Pharmacometabolomics investigates the associations between differences in metabolites and interindividual variances in drug response. Metabolomic profiling can also be used to understand disease pathophysiology and medication mechanism of action. Although promising, pharmacometabolomic studies in psychiatry are very limited. However, the formation of expert groups, such as the Pharmacometabolomics Research Network, suggests we should anticipate findings connecting metabolic profiles to drug response in the future. In fact, the network has already published several studies identifying genetic and metabolic variants relevant to psychotropic drug response (reviewed in Kaddurah-Daouk, Weinshilboum, & Network [2015]). For example, metabolomic profiling in 290 patients with MDD before and after citalopram/escitalopram treatment showed pathway activation related to tryptophan, tyrosine, and purine metabolism. Interestingly, the study also found differences in metabolites related to gut-microbiota for patients who responded and those who did not after citalopram/escitalopram treatment (Bhattacharyya et al., 2019), suggesting opportunities for integration of pharmacometabolomics and pharmacomicrobiomics.

Accounting for and integrating non-omic information into medication selection and dosing decision-making tools will also be critical to the growth of PGx-guided prescribing in psychiatry. Desirable and undesirable responses to psychotropic medications also vary by non-genomic information, such as age, sex, renal/hepatic functioning, inflammation, concomitant medications, and lifestyle factors (smoking, diet) (reviewed by Klomp, Manson, Guchelaar, & Swen, [2020]; Shah & Smith [2015]). These factors have been shown to modulate expected associations between a genomic-derived factor (e.g. metabolizer status) and medication treatment outcomes through a phenomenon by which a person's genomic-predicted phenotype does not match their clinically observed phenotype. This so-called phenoconversion has been shown to occur frequently in people receiving medications for psychiatric disorders (Mostafa, Kirkpatrick, Byron, & Sheffield, 2019; Preskorn et al., 2013), but is often not accounted for when interpreting genomic data (Bousman et al., 2022a). Notably, free phenoconversion tools have been developed to assist laboratories and healthcare providers (Bousman, Wu, Aitchison, & Cheng, 2021; Cicali et al., 2021), although these tools are focused on adjusting genomic-based phenotypes for concomitant medications only. Studies to date have primarily relied on single biomarker interactions with outcomes and do not take multiple factors into account, potentially leading to sub-clinical effects of single markers. The introduction of machine learning algorithms allows for the combination of multiple markers and clinical information to inform medication decisions and predict outcomes by utilizing a greater breadth of information beyond a single marker–drug interaction. A recent review of machine learning applications to antidepressant response showed high predictive accuracies could be obtained but independent external validation was often not performed and when it was, the predictive accuracies of the machine learning algorithms were significantly attenuated (Bobo, Van Ommeren, & Athreya, 2022). As the evidence strengthens, these tools will evolve to include a wider range of personal and environmental factors that in combination with a greater depth of -omic data will improve psychotropic medication selection and dosing decisions.

## Conclusion

In a short span of time, PGx-guided prescribing in psychiatry has emerged as an evidence-based strategy worthy of a spot in the psychiatry toolbox. Importantly, PGx-guided prescribing will not replace current prescribing strategies (e.g. therapeutic drug monitoring, hepatic/renal function testing, clinical practice guidelines), but it has and will continue to enhance selection and dosing of certain psychotropic medications. For PGx to fully transform prescribing practices, focus must turn to integrative approaches in which PGx data are combined with other -omic, personal, and environmental data to predict psychotropic medication response. To our knowledge, this approach has not been employed but with the recent emergence of powerful machine learning and artificial intelligence methods along with the global shift toward electronic health records, we anticipate example applications of such integration in the coming years. To facilitate this transformation, psychotropic clinical trials must collect and biobank specimens that will enable multi-omic interrogation of medication efficacy and tolerability. With these data in hand, it is reasonable to anticipate an expansion of clinical indications and global uptake of PGx-guided prescribing into routine clinical care.
